# Prevalence and Risk Factors Associated with the Recurrence of Infantile Hemangiomas After Discontinuation of Propranolol: A Systematic Review and Meta-Analysis

**DOI:** 10.3390/jcm14217846

**Published:** 2025-11-05

**Authors:** Chenchen Gong, Xiaojie Yue, Lulu Zhang, Xiong Zhao, Qiang Shu

**Affiliations:** 1 Department of Burns and Plastic Surgery, Children’s Hospital, School of Medicine, Zhejiang University, National Clinical Research Center for Child Health, Hangzhou 310052, China; 6513029@zju.edu.cn (L.Z.); 6196001@zju.edu.cn (X.Z.); 2 Department of Thoracic and Cardiovascular Surgery, Children’s Hospital, School of Medicine, Zhejiang University, National Clinical Research Center for Child Health, Hangzhou 310052, China; shuqiang@zju.edu.cn

**Keywords:** infantile hemangiomas, meta-analysis, propranolol, recurrence, risk factors

## Abstract

**Purpose:** The recurrence rate and related risk factors of infantile hemangiomas after treatment discontinuation remain concerns. We aim to evaluate the risk of recurrence after termination of oral propranolol for IHs and its associated risk factors. **Methods:** The Embase, PubMed, Web of Science, and Cochrane Central databases and clinicaltrials.gov were searched comprehensively for relevant studies from the inception of this study to November, 2024. Two independent reviewers conducted the data extraction and quality assessment. This review protocol was registered in the PROSPERO database (CRD42024589110). **Results:** A total of 1662 patients in 10 studies met the eligibility criteria, which was predominantly retrospective in design. All participants were infants diagnosed with infantile hemangiomas who received oral propranolol therapy; the majority of patients received propranolol treatment for at least six months. The results revealed that the pooled recurrence rate was 20% (95% CI: 15–24%), and 11% of patients required retreatment with propranolol (95% CI: 9–14%). Female sex (OR = 1.76, 95% CI: 1.20–2.59) and IHs located on the head and neck (OR = 2.40, 95% CI 1.59–3.63) increased the risk of recurrence. In contrast, IH type, lesion distribution, duration of therapy, and treatment initiation age showed no significant associations. Additionally, one trial included in this review reported that continued medication for one month after the lesion reaches its maximum degree of regression might increase the risk of recurrence as compared to three months of maintenance (OR = 1.86, 95% CI 0.98–3.5); however, the evidence is limited and preliminary. **Conclusions:** Female sex and IHs located on the neck or head contribute to the recurrence of IHs after termination of treatment. In addition, the type of IH and withdrawal criteria may influence recurrence risk, although evidence remains limited. Thus, optimizing treatment protocols, including individualized therapy duration and discontinuation strategies, may help reduce recurrence rates.

## 1. Introduction

Infantile hemangiomas (IHs) are the most frequent benign tumors in infancy, with an incidence of 4–10% [1]. Their growth cycle consists of three stages: the early and late proliferative stages, followed by the slow involution phase [1]. While most of them resolve spontaneously, a subset of IHs can cause serious complications (like organ dysfunction or even life-threatening outcomes) and cosmetic disfigurement [2,3]. Thus, it is necessary to make rational and effective treatment strategies.

Over the past two decades, propranolol has become the first-line therapy for IHs requiring treatment [4,5]. With the widespread use of propranolol, the recurrence rate after treatment cessation and its risk factors have been of great concern, but these issues have not been completely elucidated [6,7]. Although several cohort studies have investigated recurrence, the reported incidences vary considerably. For example, Bonifazi E et al. [8]. reported a 29% recurrence rate during a 3-month follow-up, whereas L. G. Mariani et al. [9] reported a recurrence rate of 15% over an average follow-up of 11 months.

Recently, several studies have attempted to clarify the potential risk factors for recurrence after termination of propranolol. For instance, limb-located IHs were found to have a significantly lower relapse rate than lesions in the head and neck region [10]. Additionally, Luying Wang et al. reported that maintaining propranolol for three months after achieving maximal regression significantly reduced the risk of major relapse compared with a one-month maintenance period [11].

Despite these findings, the overall evidence remains limited and fragmented. Therefore, this meta-analysis aims to systematically estimate the recurrence rate and identify potential determinants among IHs treated with oral propranolol, thereby providing more robust evidence to guide clinical follow-up and individualized management strategies.

## 2. Methods

This meta-analysis was performed according to the Cochrane Handbook for Systematic Reviews of Interventions and presented in accordance with Preferred Reporting Items for Systematic Reviews and Meta-analyses guidelines (PRISMA Guidelines). The study protocol was registered with the International Prospective Register of Systematic Reviews (PROSPERO CRD42024589110).

### 2.1. Search Strategy

A systematic literature review was performed on the Embase, PubMed, Web of Science, Cochrane Central databases and clinicaltrials.gov for relevant studies from the inception of this study to November, 2024. A basic search was carried out using the keywords as follows: “hemangioma”, “propranolol”, and “recurrence”. The detailed search strategy is available in Data S1. Reference lists of relevant sources were hand-searched and enquiries were made to content experts for relevant studies. Abstracts retrieved from the electronic databases along with single additional articles were subsequently imported into EndNote 20 (Clarivate, Philadelphia, PA, USA) for the removal of duplicates. This process was conducted independently by C.G. and L.Z. Any disparities were resolved by consensus (X.Y.).

### 2.2. Eligibility Criteria

Trials were selected based on the following inclusion criteria: (1) the included participants were treated with oral propranolol alone and completed the course of treatment; (2) original articles encompassing randomized controlled trials (RCTs), cohort, and cross-sectional studies; (3) written in or translated to the English language. Studies were excluded if (1) there was no primary data (meta-analyses, literature reviews, protocols, letters, commentaries, and editorials); (2) they had a sample size of 10 or less; (3) overlapping datasets were used; or (4) data could not be extracted from their studies.

### 2.3. Data Extraction and Quality Assessment

Two authors (C.G., L.Z.) independently extracted data on general study information (first author, year of publication, country, and sample size), baseline demographic and clinical characteristics (age, gender, follow-up duration, and the definition of recurrence), and interventions (administration for oral propranolol number of patients receiving oral propranolol), as well as outcomes of interest.

For quality assessments, the Newcastle–Ottawa Scale (NOS) for cohort studies [12] and Cochrane risk of bias criteria for RCT were used. Studies with an NOS score of 7 or more were considered high quality. Disagreements on data extraction and quality assessment between the 2 reviewers were resolved by consensus (X.Z., Q.S.).

### 2.4. Outcomes

The primary outcome was the incidence of recurrent IH (increased IH color, surface/volume, or texture after oral propranolol cessation) during follow-up, while the secondary outcome collected was the major relapse (referring to significant changes in color, surface/volume, or texture requiring a second course of oral propranolol) rate after discontinuation of propranolol. The definition of recurrence in different studies is displayed in Table S1.

For each risk factor, we extracted the ORs or RRs, together with 95% CI. Multivariate estimates were always selected when available; otherwise, the unadjusted results were recorded. When the OR was not provided, a crude OR would be computed if possible. There was no restriction on the follow-up length. Conflicts were arbitrated through a group discussion including the senior author (X.Y.).

### 2.5. Statistical Analysis

Meta-analyses of proportions were conducted for the incidence of recurrence, as well as the prevalence of major recurrence. We presented the risk factors by calculating odds ratios (ORs) and 95% CIs; data analyses were performed using Stata 17. Study heterogeneity was conducted using Cochran’s Q statistic and I^2^ statistics; *p* < 0.5 for the former and a value > 50% for the latter were assumed to indicate significance and high heterogeneity [13,14]. If high heterogeneity existed, pooling of effect sizes was performed using a random-effects model; otherwise, a fixed-effects model was used [14]. Additionally, we planned to assess reporting bias (risk of bias due to missing results) following guidance in the Cochrane Handbook (Chapter 13). Funnel plot asymmetry and statistical tests were to be conducted if at least 10 studies were available for an outcome.

In addition, subgroup analysis was conducted using the following variables to explore the sources of heterogeneity: the incidence of recurrence; location; sex; sample size; and data type (retrospective data or prospective data). Moreover, sensitivity analyses were conducted using a leave-one-out approach, whereby the pooled effect was recalculated after sequentially omitting each included study to assess the influence of individual studies on the overall estimate.

## 3. Results

### 3.1. Retrieving Identified Studies

**Figure 1:**
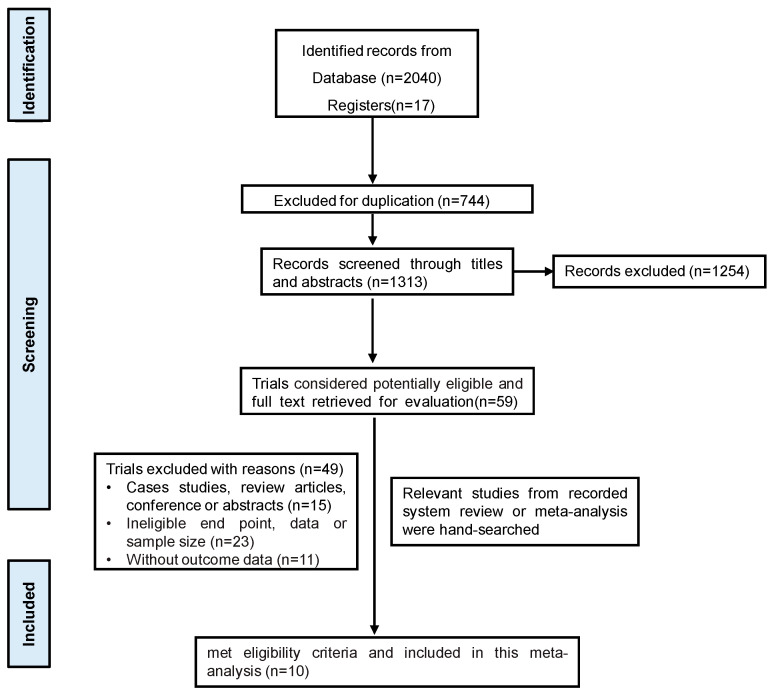
The flow diagram of study selection.

Through study retrieval, a total of 2057 studies were finally included. Following the review of the titles and abstracts, 59 studies were deemed appropriate and were eligible for potential inclusion. After full-text review, 10 studies were finally included (Figure 1).

### 3.2. Description of the Included Studies

The 10 articles comprised 9 cohort studies and a single RCT. All observational studies achieved a moderate or high quality score of between 6 and 9 on the NOS Assessment Form. Key study characteristics, quality assessments, patient demographics, and clinical variables of the patient population are detailed in Table 1 and Table 2.

### 3.3. Outcomes of the Pooled Studies

#### 3.3.1. Primary Outcomes

**Figure 2:**
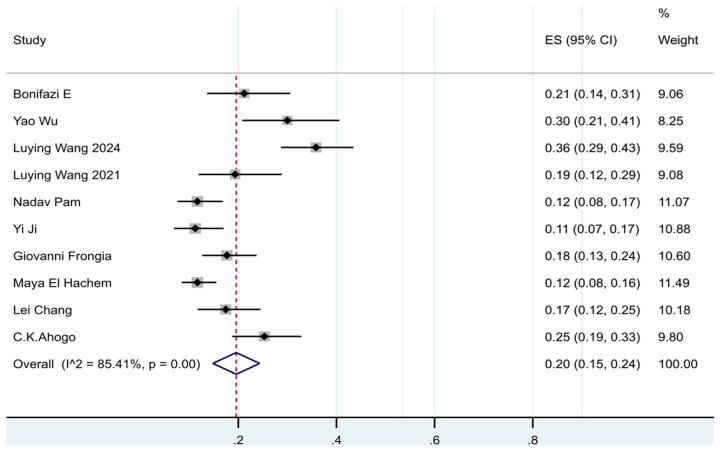
Forest plot of the prevalence of recurrence in patients with IHs after termination of propranolol.

Of the 10 [7,8,11,15,16,17,18,19,20,21] studies that assessed the IH recurrence rate after discontinuation of oral propranolol in the meta-analysis, according to the results of the meta-analysis, the pooled prevalence was 20% (95% CI: 15–24%, I^2^ = 85%, *p* < 0.01) (See Figure 2).

To explore the sources of heterogeneity, this study further performed subgroup analyses of recurrence rate. The estimates of pooled prevalence of recurrence for Asia and European were 20% and 18%, respectively. The estimates of pooled prevalence of recurrence were 25% in females and 16% in males. The median sample size of the included studies was calculated to be 167, so the studies with a sample size less than 167 were named as small sample studies, and those with a sample size ≥ 167 were named as large sample studies. The prevalence of recurrence was 25% in the small sample group and 13% in the large sample group. The prevalence of recurrence data obtained through prospective studies was 21% and through retrospective studies was 19%. A formal subgroup analysis according to follow-up duration was not feasible because included studies reported follow-up inconsistently—some defined it as post-withdrawal observation, while others reported the total period including treatment. The estimated pooled results obtained in subgroup analyses were shown in Supplementary Figures S1–S4, and the results indicated that the sample size was the main source of heterogeneity.

In addition, sensitivity analyses, performed by sequentially omitting individual studies, indicated that the overall recurrence rate remained stable, supporting the robustness of the findings (See Table S2).

#### 3.3.2. Secondary Outcomes

**Figure 3:**
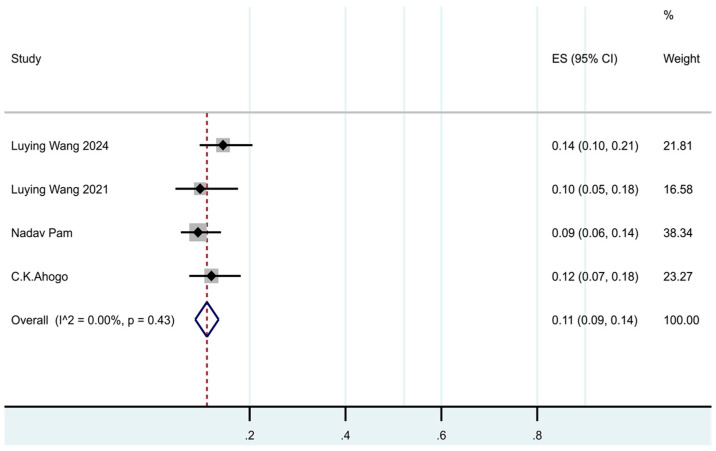
Forest plot of the prevalence of major recurrence in patients with IHs after termination of propranolol.

In our meta-analysis, 4 trials [7,11,16,17] included major relapse data; the pooled major relapse rate was 11% (95% CI: 9–14%, I^2^ = 0%, *p* = 0.43). (See Figure 3.)

### 3.4. Risk Factors of Recurrence After Termination of Oral Propranolol

#### 3.4.1. Patient-Related Factors

Gender (female versus male)

**Figure 4:**
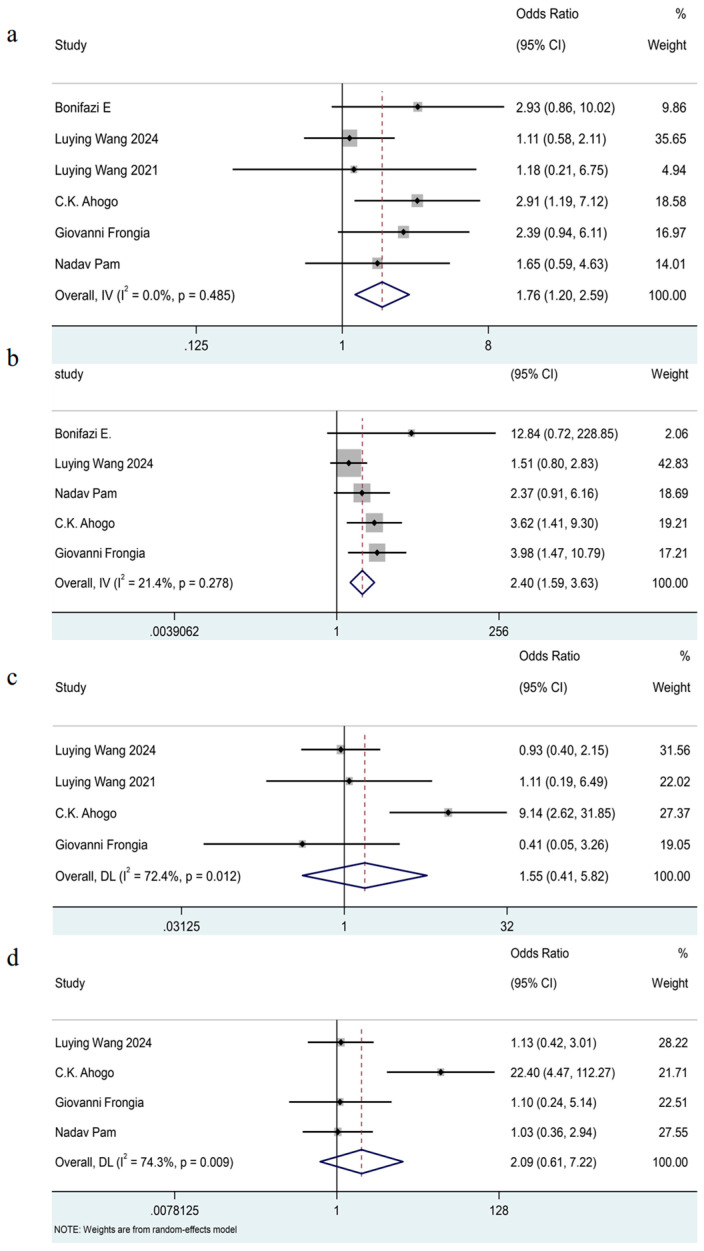
Forest plot detailing the association of patient-related factors as dichotomous variables with recurrence in patients with IHs after termination of propranolol. (**a**) Gender (female versus male); (**b**) the sites of IHs (head and neck versus others); (**c**) the distribution of IHs (segmental versus nonsegmental); (**d**) the type of IHs (deep versus superficial).

The results of the pooled analysis [7,8,11,16,17,19] showed that females had significantly increased odds of recurrence compared with males (OR = 1.76, 95% CI: 1.20–2.59, I^2^ = 0%, *p* = 0.49). (See Figure 4a.) As all included estimates were unadjusted, this finding should be interpreted with caution.

The sites of IHs (head and neck versus others)

The meta-analysis included five trials [7,8,11,17,19] which showed the IHs located on the head and neck exhibited a 2.40-fold risk for recurrence rate compared to those in other regions (OR = 2.40, 95% CI 1.59–3.63, I^2^ = 21%, *p* = 0.28). (See Figure 4b.)

Furthermore, a pooled of two studies indicated that the regrowth rates in the facial region [8,19] were significantly high compared with other sites (OR = 3.48, 95% CI 1.65–7.36, I^2^ = 0%, *p* = 0.98). (See Supplementary Figure S5.)

The distribution of IHs (segmental versus nonsegmental)

The pooled analysis included four studies [7,11,16,19] and showed that there was no significant association between the distribution of IHs and their recurrence (OR = 1.55, 95% CI 0.41–5.82, I^2^ = 72%, *p* = 0.01). (See Figure 4c.)

The type of IHs (deep versus superficial)

The meta-analysis included four trials [7,11,17,19] and indicated that there was no significant association between the type of IHs and their recurrence after termination of oral propranolol (OR = 2.09, 95% CI 0.61–7.22, I^2^ = 74%, *p* < 0.01). (See Figure 4d.)

Given the substantial heterogeneity, we conducted a leave-one-out sensitivity analysis to explore the potential source of heterogeneity (see Table S3). The analysis identified a study by C.K. Ahogo [7] as having a considerable impact on the heterogeneity. Further investigation revealed that most patients with IHs in this study received treatment for less than six months; additionally, sample characteristics exhibited differences (like age at treatment initiation and the proportion of IHs patients with a deep component among the total participants), which may account for its influence. Nonetheless, the overall direction of the effect remained consistent with or without this study, suggesting the robustness of the findings.

#### 3.4.2. Treatment-Related Factors

Age at Treatment Initiation (Later Treatment Initiation Versus Earlier Treatment Initiation)

**Figure 5:**
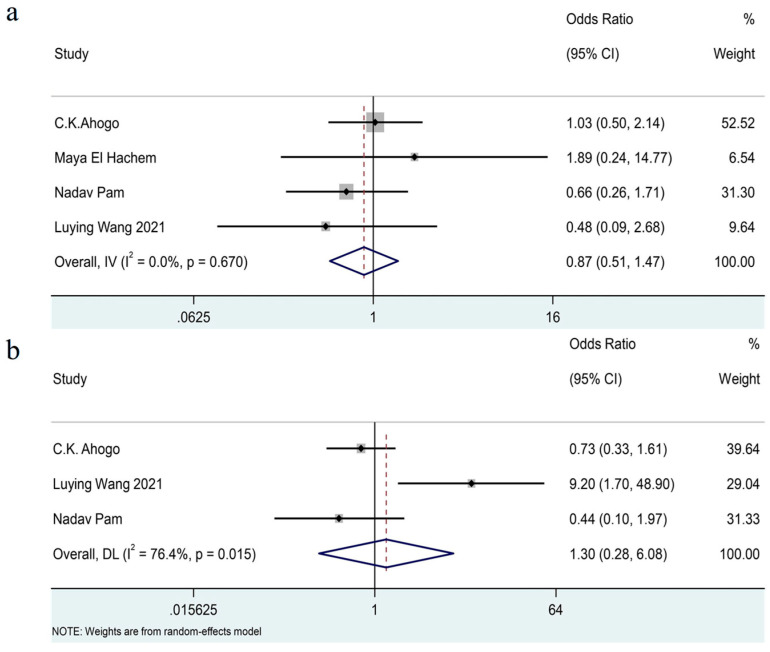
Forest plot detailing the association of age at treatment initiation (**a**) and treatment duration (**b**) as dichotomous variables with recurrence in patients with IHs after termination of propranolol.

Due to inconsistencies in the cutoff points for early and late treatment initiation across studies, we defined this variable as a binary category (later treatment initiation versus earlier treatment initiation). The pooled outcomes [7,16,17,20] showed that there was no significant association of the age at treatment initiation with recurrence (OR = 0.87, 95% CI 0.51–1.47, I^2^ = 0%, *p* = 0.67). (See Figure 5a).

The Duration of Treatment (≤6 Months Versus >6 Months)

The pooled analysis [7,16,17] included three studies and showed that there was no significant association between the duration of treatment and recurrence (OR = 1.30, 95% CI 0.28–6.08, I^2^ = 76%, *p* = 0.02). (See Figure 5b.)

*The Withdrawal Criteria (Direct Withdrawal Versus Gradual Withdrawal and Withdrawal with 1-Month Maintenance Versus with 3-Month Maintenance)*

One included study [11] showed that, as compared with direct withdrawal, gradual withdrawal did not significantly influence the recurrence rate. But continued medication for 1 month after the lesion reached its maximum degree of regression might increase the risk of relapse compared with 3 months of maintenance (OR = 1.86, 95% CI 0.98–3.5, *p* = 0.06), especially for increasing the risk of major relapse (OR = 2.57, 95% CI 1.01–6.52, *p* = 0.04).

### 3.5. The Risk of Bias Due to Missed Results

For each outcome, fewer than 10 studies were available; therefore, we did not perform formal tests for funnel plot asymmetry. Nevertheless, we acknowledge the potential risk of reporting bias and selective outcome reporting, which may have influenced the findings.

## 4. Discussion

In this systematic review and meta-analysis, the recurrence rate of IHs varied from 15% to 24%, consistent with previous reports in the literature [6,22,23]. Moreover, 11% of patients had a major relapse requiring a second course of propranolol. This suggests that while propranolol is highly effective in treating IHs, a significant proportion of patients experience recurrence after treatment cessation. Thus, investigating risk factors for recurrence after discontinuation of propranolol helps pediatricians and patients coordinate an optimal pharmacological treatment strategy.

Several risk factors were identified as being significantly associated with IHs recurrence. In this meta-analysis, female gender conferred an increased risk of recurrence (OR = 1.76). This observation may be related to estrogen-mediated effects on vascular endothelial growth factor expression, which could potentially promote endothelial cell proliferation; however, this mechanism remains exploratory and is based on limited evidence [24,25]. Moreover, since these results were derived from unadjusted estimates, they should be interpreted with caution. Potential confounding factors—such as lesion depth, treatment duration, and disease severity—might have influenced this association, underscoring the need for further studies with multivariate analyses.

Additionally, anatomical location emerged as one of the crucial determinants, with IHs located on the head and neck exhibiting a 2.40-fold risk for recurrence compared to those in other regions. Notably, recurrence rates in the facial region were significantly higher than in other sites (OR = 3.48). This finding aligns with previous studies suggesting that IHs localized on the lower body have a tendency to show minimal or arrested growth compared with those on the upper body [26,27]. Moreover, one of enrolled studies reported that IHs with a deep component had significantly increased odds of recurrence compared with superficial IHs, but the meta-analysis did not identify a significant association between lesion depth and recurrence. This finding cannot be considered conclusive given the limited number of studies, substantial heterogeneity, and variations in patient characteristics and treatment duration. While previous studies have suggested that deeper lesions may have a longer proliferative phase and thus a greater tendency toward recurrence [6,24,28], the current evidence from our meta-analysis remains inconclusive. Nevertheless, clinicians may consider closer follow-up for deep IHs in complex cases until stronger evidence becomes available.

In addition, we found that the age at which propranolol treatment was initiated did not affect the recurrence, but early treatment initiation has been associated with improved outcomes, suggesting that timely intervention may enhance the long-term efficacy of propranolol [15,29]. Moreover, in this meta-analysis, there was no significant association between the duration of treatment and the recurrence; similar results were observed in the studies by Shah et al. [6] and C. Mauguen et al. [24]. Of course, these non-significant findings may reflect limited statistical power rather than a true lack of effect. The relatively small number of included studies, modest sample sizes, and considerable heterogeneity in study design and outcome definitions could have diminished the ability to detect subtle but clinically relevant associations. More importantly, the age at which treatment is discontinued, as a proxy for the stage of IHs, may have a greater impact on the risk of relapse than the treatment duration [24], which might be due to a prolonged proliferative phase or a higher risk of delayed recurrence [30,31,32]. Thus, an appropriate discontinuation protocol may play an important role in minimizing the recurrence rate after the withdrawal of propranolol treatment.

At present, few studies focus on the optimal strategies to discontinue propranolol treatment in IHs. In our systematic review, only one of the included studies [11] discussed withdrawal criteria; it reported that, compared with direct withdrawal, gradual withdrawal (halving the dosage per week and discontinuing medication within 4 weeks) did not reduce the risk of recurrence or major relapse, which was in line with the previous studies [6,24]. However, 3 months of maintenance after the lesion reached its maximum degree of regression was helpful in reducing the risk of recurrence, especially when withdrawal occurred for patients with age older than 13 months who received 3 months of maintenance [11]. The validity of this withdrawal mode needs to be tested in larger sample sizes and higher-quality studies.

In addition to patient-related and treatment-related factors, complex vascular syndromes such as PHACE(S) may influence recurrence risk. These syndromes often involve large or segmental hemangiomas associated with arterial, cardiac, or cerebral anomalies. According to recent study [33], patients with PHACE(S) may have incomplete regression and a higher likelihood of rebound due to extensive vascular abnormalities and residual proliferative tissue. Therefore, individualized treatment duration and close post-treatment monitoring are recommended in this subgroup.

Moreover, systemic inflammatory or infectious conditions in early childhood might affect hemangioma behavior and therapeutic response. Although direct evidence linking infection to recurrence is limited, it is possible that systemic inflammation may disrupt endothelial homeostasis [34,35], potentially altering angiogenic pathways and affecting treatment efficacy, which could contribute to rebound growth in predisposed infants. This potential link warrants further investigation in prospective clinical or mechanistic studies.

While reinitiating propranolol remains effective for most recurrent IHs [11,16,18,36], emerging strategies targeting underlying molecular pathways offer promise. Elevated miRNA levels in recurrent lesions suggest that combining propranolol with miRNA inhibitors may enhance therapeutic durability [37]. Additionally, statins have shown anti-angiogenic effects in preclinical IH models, suggesting a potential role as adjunctive agents to prevent relapse; however, these findings are preliminary and require further validation in clinical studies [38]. Moreover, several plant-derived compounds (e.g., 15,16-dihydrotanshinone I from *Salvia miltiorrhiza*, proanthocyanidins, and lingonberry extracts) have shown anti-angiogenic or antiproliferative effects in preclinical studies [39], with DHTS reportedly more effective than propranolol [40], but their use in IHs has not been tested in humans, and their safety, dosage, and pharmacokinetics in infants remain unknown. Thus, these compounds are of potential interest but cannot currently be recommended for IH treatment.

This meta-analysis is limited by small sample sizes, retrospective study designs susceptible to bias, and considerable heterogeneity in patient profiles and follow-up durations, which may compromise the robustness and generalizability of the results. This meta-analysis has several limitations. The high heterogeneity among studies may weaken the reliability of the results (such as pooled recurrence estimates). In addition, restricting inclusion to English-language publications and excluding studies with fewer than 10 participants may have introduced publication and selection bias. These factors should be considered when interpreting the results.

## 5. Conclusions

In conclusion, this meta-analysis revealed that approximately 20% of infantile hemangiomas recur after propranolol withdrawal. Female sex and head and neck lesions were associated with a higher recurrence risk, whereas lesion type, distribution, treatment duration, and age at initiation showed no significant associations—likely due to sample limitations and interstudy heterogeneity. Differences in withdrawal criteria may also contribute to outcome variability. These findings highlight the need for standardized yet individualized treatment and discontinuation protocols, along with appropriate post-treatment follow-up. Future large-scale prospective studies are warranted to confirm these associations and guide optimized management strategies. 

## Figures and Tables

**Table 1:** Study details, baseline demographic and clinical characteristics.

Author	Country	Year	Study Design	Sample Size (N)	Female (%)	Age (Months) (Mean (SD) or Median/Mean (IQR))	Follow-Up Length (Months)	Quality Assessment ^&^
Bonifazi E [8]	Italy	2014	cohort	99	70.0	mean: 4.3, range:1–19	3 months after discontinuation	6
Yao Wu [15]	China	2023	cohort	90	72.3	group 1: 4.3 (2.97); group 2: 5.2 (4.57)	range: 5–52	7
Luying Wang [11]	China	2024	cohort	150	62.7	group A: 2.80 (2.05–4.40), group B: 3.50 (2.20–4.40), group C: 3.85 (1.73–5.55), group D: 2.70 (2.30–5.20)	6 months after discontinuation	8
Luying Wang [16]	China	2021	cohort	100	71.0	group A: median: 3.5, group B: median: 3.7	9 months after discontinuation	9
Nadav Pam [17]	Israel	2021	cohort	206	70.9	4.8 (3.1)	4 months after discontinuation	8
Yi Ji [18]	China	2021	RCT	169	76.8	2.55 (1.0)	up to 24	low-risk ^#^
Giovanni Frongia [19]	Germany	2021	cohort	198	75.0	2 (2–4)	median (IQR): 8.5 (7–12.9)	7
Maya El Hachem [20]	Italy	2017	cohort	343	70.8	group 1: median: 0.8, range: 0–1.1, group 2: median: 9, range 5–90.7	≥1 month after discontinuation	7
Lei Chang [21]	China	2017	cohort	149	72.4	group 1: 3.1 (1.2), group 2: 3.4 (1.3)	range: 6–25	6
C.K. Ahogo [7]	France	2013	cohort	158	67.1	≤5	>6 months after discontinuation	9

IQR, interquartile range; RCT, randomized controlled trial; SD, standard deviation. ^&^ Newcastle–Ottawa Scale (NOS) for non-randomized cohort and case–control studies in meta-analysis (0–3: high risk of bias; 4–6: moderate risk of bias; 7–9: low risk of bias). ^#^ Cochrane risk of bias criteria for RCT study quality assessment: randomization sequence generation, allocation concealment, blinding of participants and personnel, blinding of outcome assessment, incomplete outcome data, selective reporting, and other biases are all assessed as low-risk.

**Table 2:** Recurrence rates and related risk factors of infantile hemangiomas.

Study (Author, Year)	Recurrence Rate	Major Recurrence Rate	Potential Risk Factor (s)
Bonifazi E, 2014 [8]	21/99	NA	gender, lesion site
Yao Wu, 2023 [15]	27/90	NA	NA
Luying Wang, 2024 [11]	62/173 *	25/173 *	gender, lesion site, type of hemangioma, distribution of hemangioma, age at treatment initiation, withdrawal criterion for discontinuation
Luying Wang, 2021 [16]	18/93 ^$^	9/93 ^$^	gender, type of hemangioma, distribution of hemangioma, age at treatment initiation, duration of treatment
Nadav Pam, 2021 [17]	24/206	19/206	gender, lesion site, type of hemangioma, age at treatment initiation, duration of treatment
Yi Ji, 2021 [18]	19/169	NA	NA
Giovanni Frongia, 2021 [19]	35/198	NA	gender, lesion site, type of hemangioma, distribution of hemangioma
Maya El Hachem, 2017 [20]	40/343	NA	NA
Lei Chang, 2017 [21]	26/149	NA	NA
C.K. Ahogo, 2013 [7]	40/158	19/158	gender, lesion site, type of hemangioma, distribution of hemangioma, age at treatment initiation, duration of treatment

NA, data not available. * Represents 150 patients with 173 lesions. ^$^ In total, 7 patients lost to follow-up by 9 months after termination of treatment.

## Supplementary Materials

The following supporting information can be downloaded at: https://www.mdpi.com/article/10.3390/jcm14217846/s1, 1. Figure and table supplemental: Table S1: The definition of recurrence in included studies. Table S2: Leave-One-Out Sensitivity: analysis of the robustness of pooled recurrence rate. Table S3: Leave-One-Out Sensitivity: analysis of the association between type of IHs and recurrence rate. Figure S1: Forest plot of subgroup analysis of the prevalence of recurrence in patients with IHs after termination of Propranolol based on study location. Figure S2: Forest plot of Subgroup analysis of the prevalence of recurrence in patients with IHs after termination of Propranolol based on gender. Figure S3: Forest plot of Subgroup analysis of the prevalence of recurrence in patients with IHs after termination of Propranolol based on sample size. Figure S4: Forest plot of Subgroup analysis of the prevalence of recurrence in patients with IHs after termination of Propranolol based on data type. Figure S5: Forest plot detailing the association of IHs in the facial regions as dichotomous variables with recurrence in patients with IHs after termination of Propranolol. 2. Data S1 search strategy. 3. PRISMA_2020_checklist [41].
