# Supplementary material for: Prevalence and Risk Factors Associated with the Recurrence of Infantile Hemangiomas After Discontinuation of Propranolol: A Systematic Review and Meta-Analysis
Source: J Clin Med. 2025 Nov 5;14(21):7846. doi: 10.3390/jcm14217846 (PMC12607970; doi:10.3390/jcm14217846)
Supplement: Supplementary file 1 [file jcm-14-07846-s001.zip › Figure Supplemental.docx]

**Legends:**

**Table S1** The definition of recurrence in included studies.

**Table S2** Leave-One-Out Sensitivity: analysis of the robustness of pooled recurrence rate.

**Table S3:** Leave-One-Out Sensitivity: analysis of the association between type of IHs and recurrence rate.

**Figure S1** Forest plot of subgroup analysis of the prevalence of recurrence in patients with IHs after termination of Propranolol based on study location.

**Figure S2** Forest plot of Subgroup analysis of the prevalence of recurrence in patients with IHs after termination of Propranolol based on gender.

**Figure S3** Forest plot of Subgroup analysis of the prevalence of recurrence in patients with IHs after termination of Propranolol based on sample size.

**Figure S4** Forest plot of Subgroup analysis of the prevalence of recurrence in patients with IHs after termination of Propranolol based on data type.

**Figure S5** Forest plot detailing the association of IHs in the facial regions as dichotomous variables with recurrence in patients with IHs after termination of Propranolol.

**Table S1** The definition of recurrence in included studies

| **Study (Author, Year)** | **Definition of recurrence/relapse** | **Evaluation method** |
| --- | --- | --- |
| Bonifazi E, 2014 | Increase in lesion size >20% | Clinical observation and photograph review |
| Yao Wu, 2023 | NA | NA |
| Luying Wang, 2024 | **relapse:** any degree of flushing, enlargement, or increase in blood flow.  **major relapse:** significant changes in color, volume, and blood flow, requiring further treatment | Doppler ultrasound |
| Luying Wang, 2021 | regrowth in size, worsening of color, and recurring ulceration | Clinical observation and photograph review |
| Nadav Pam, 2021 | **≥50%** expansion in the dimension of a lesion that had previously responded well to systemic propranolol. | Clinical observation and photograph review |
| Yi Ji, 2021 | Regrowth of more than **20%** in hemangioma appearance (including changes in color and/or volume) | Clinical observation |
| Giovanni Frongia, 2020 | increased IH color, surface/volume, or texture | Clinical observation |
| Maya El Hachem, 2017 | NA | NA |
| Lei Chang, 2017 | obvious regrowth of a primary lesion and abundant blood flow | Doppler ultrasound |
| C.K. Ahogo, 2013 | **minor relapse:** isolated mild recoloration of the IH that did not need retreating  **major relapse:** regrowth associated with recoloration and/or ulceration of the IH that needed to be retreated | Clinical observation and photograph review |

**Table S2** Leave-One-Out Sensitivity: Leave-One-Out Sensitivity: analysis of the robustness of pooled recurrence rate

| **Study Removed** | **Pooled OR (95% CI)** | **I² (%)** | **Change Observed** |
| --- | --- | --- | --- |
| None (All included) | 0.20 (0.15-0.24) | 85.41% | Reference |
| Bonifazi E | 0.19 (0.14–0.25) | 86.74% | I² Slight increase |
| Yao Wu | 0.19 (0.14–0.23) | 85.08% | I² Slight decrease |
| Luying Wang 2024 | 0.17 (0.14-0.21) | 74.84% | I² Slight decrease |
| Luying Wang 2021 | 0.20 (0.14-0.25) | 86.93% | I² Slight increase |
| Nadav Pam | 0.21 (0.15-0.26) | 85.74% | I² Slight increase |
| Yi Ji | 0.21 (0.15-0.26) | 85.79% | I² Slight increase |
| Giovanni Frongia | 0.20 (0.14-0.25) | 86.99% | I² Slight increase |
| Maya El Hachem | 0.21 (0.15-0.26) | 84.34% | I² Slight decrease |
| Lei Chang | 0.20 (0.15-0.25) | 87.01% | I² Slight increase |
| C.K.Ahogo | 0.19 (0.14-0.24) | 85.39% | I² Slight decrease |

**Table S3** Leave-One-Out Sensitivity Analysis: analysis of the association between type of IHs and recurrence rate

| **Study Removed** | **Pooled OR (95% CI)** | **I² (%)** | **Change Observed** |
| --- | --- | --- | --- |
| None (All included) | 2.09 (0.61–7.22) | 74% | Reference |
| Luying Wang 2024 | 2.77 (0.43–17.69) | 81% | I² Slight increase |
| C.K. Ahogo | 1.09 (0.57–2.08) | 0% | I² decreased significantly |
| Giovanni Frongia | 2.62 (0.51–13.35) | 82% | I² Slight increase |
| Nadav Pam | 2.84(0.47–17.21) | 81% | I² Slight increase |


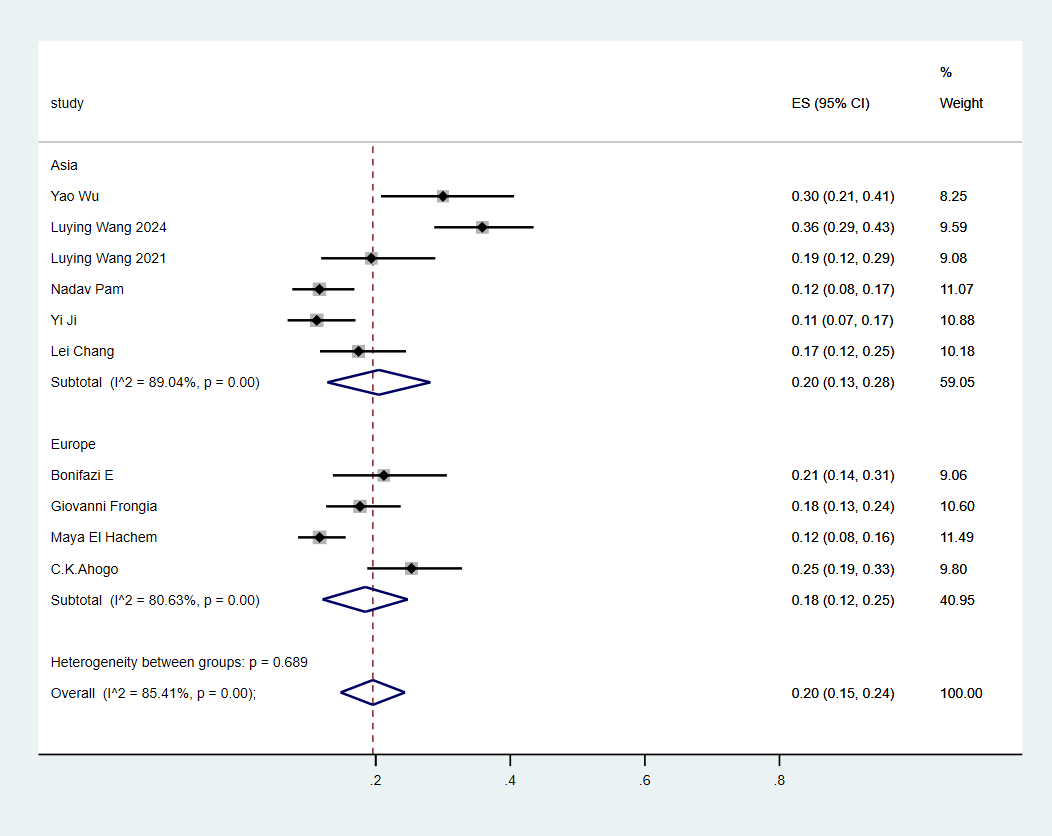


**Figure S1** Forest plot of subgroup analysis of the prevalence of recurrence in patients with IHs after termination of Propranolol based on study location.


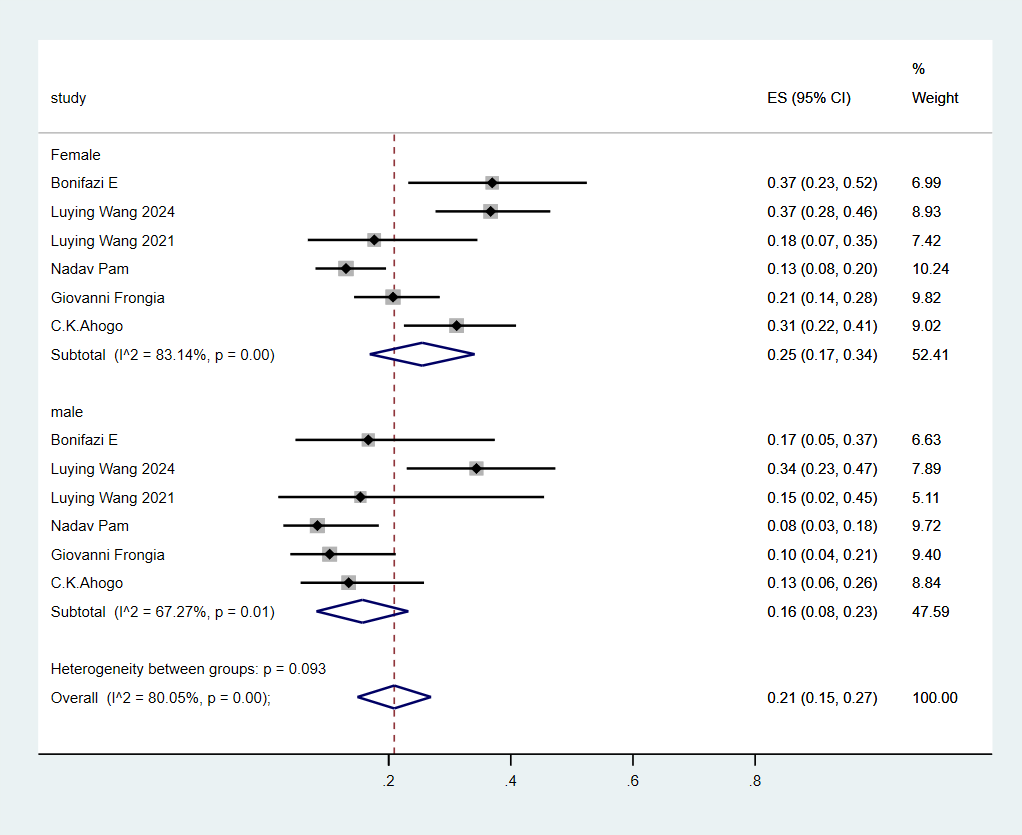


**Figure S2** Forest plot of Subgroup analysis of the prevalence of recurrence in patients with IHs after termination of Propranolol based on gender.


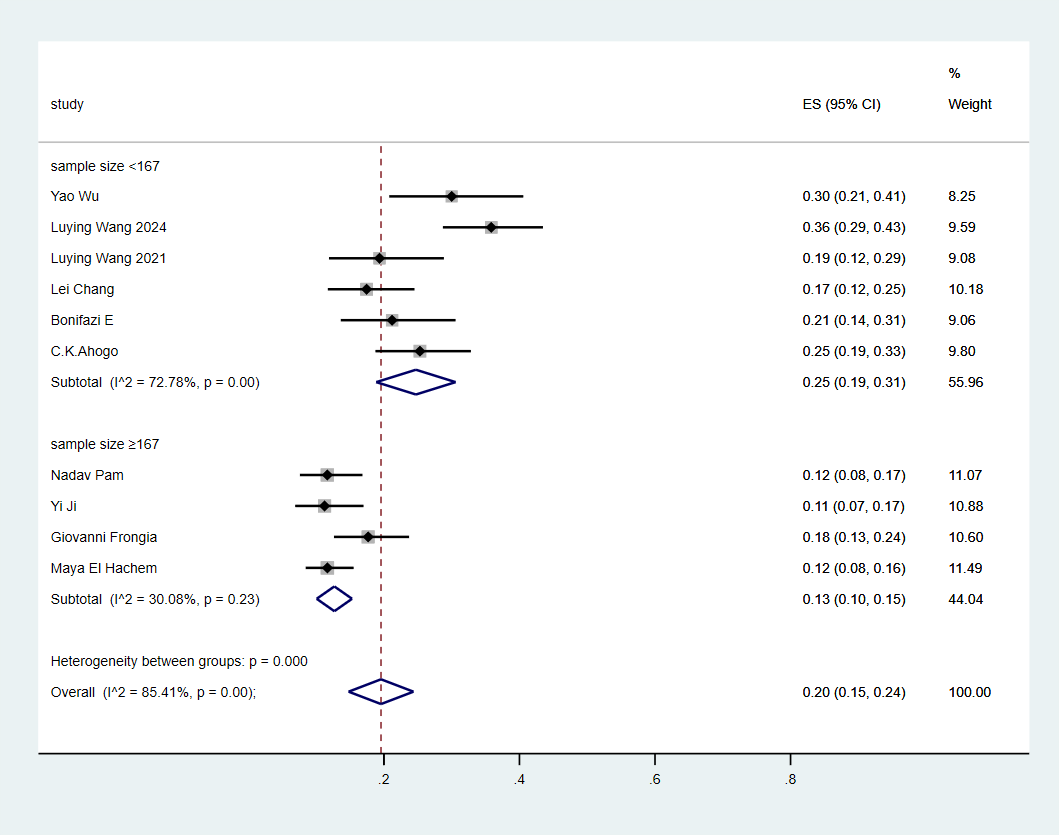


**Figure S3** Forest plot of Subgroup analysis of the prevalence of recurrence in patients with IHs after termination of Propranolol based on sample size.


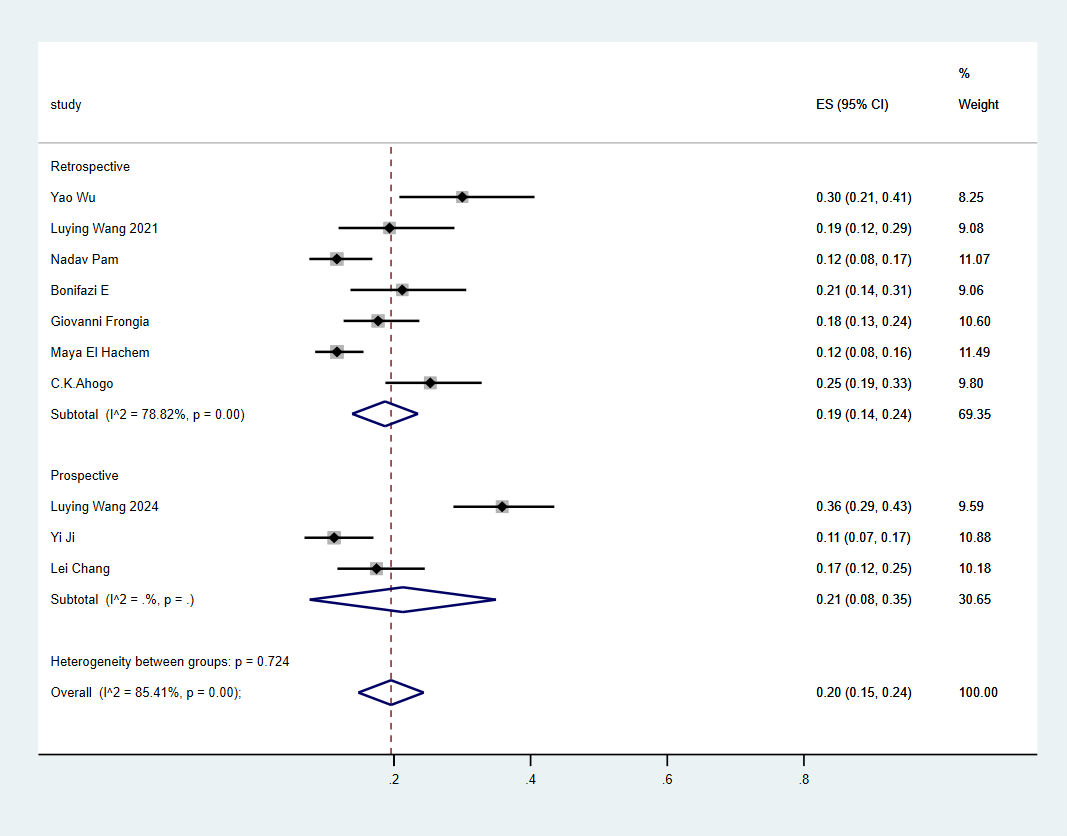


**Figure S4** Forest plot of Subgroup analysis of the prevalence of recurrence in patients with IHs after termination of Propranolol based on data type.


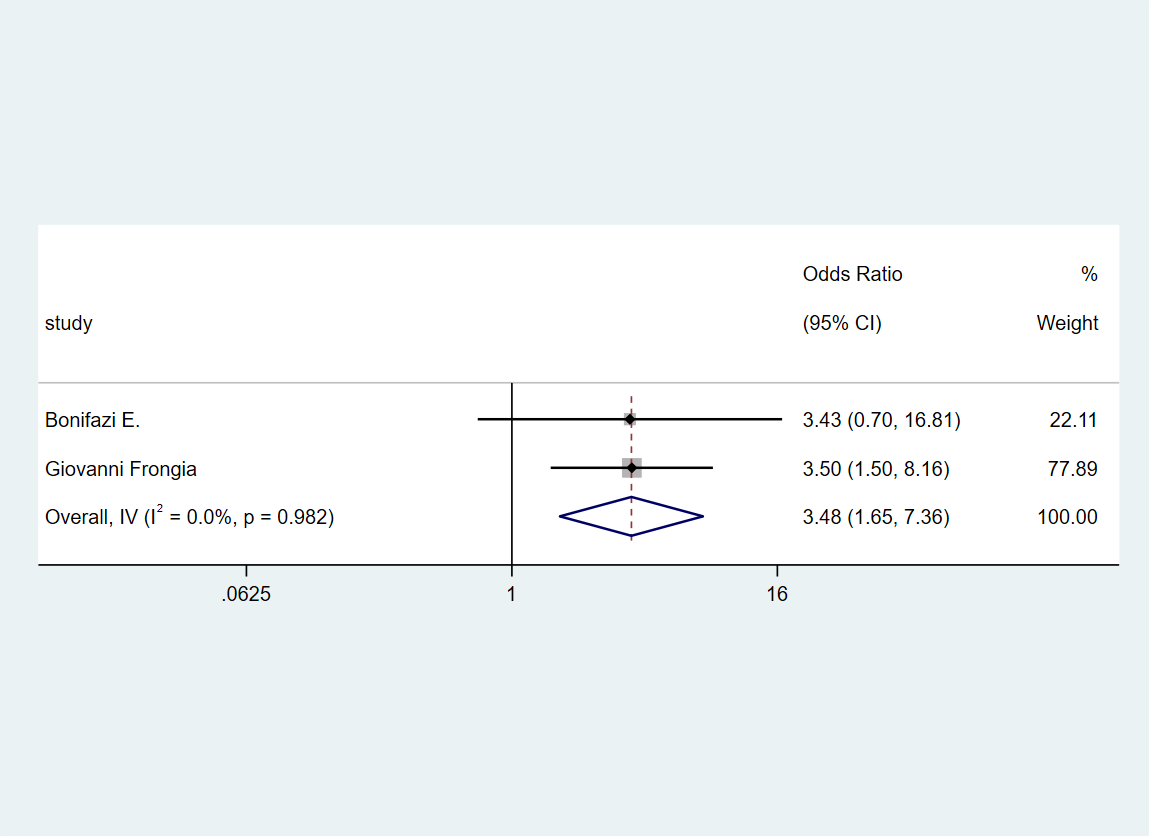


**Figure S5** Forest plot detailing the association of IHs in the facial regions as dichotomous variables with recurrence in patients with IHs after termination of Propranolol.
