# Supplementary material for: Prevalence and Risk Factors Associated with the Recurrence of Infantile Hemangiomas After Discontinuation of Propranolol: A Systematic Review and Meta-Analysis
Source: J Clin Med. 2025 Nov 5;14(21):7846. doi: 10.3390/jcm14217846 (PMC12607970; doi:10.3390/jcm14217846)
Supplement: Supplementary file 1 [file jcm-14-07846-s001.zip › jcm-3909416 - Data S1search strategy .docx]

**Search strategy：**

Cochrane Central databases（-**2024.11.18**）(49 trials+2 reviews)


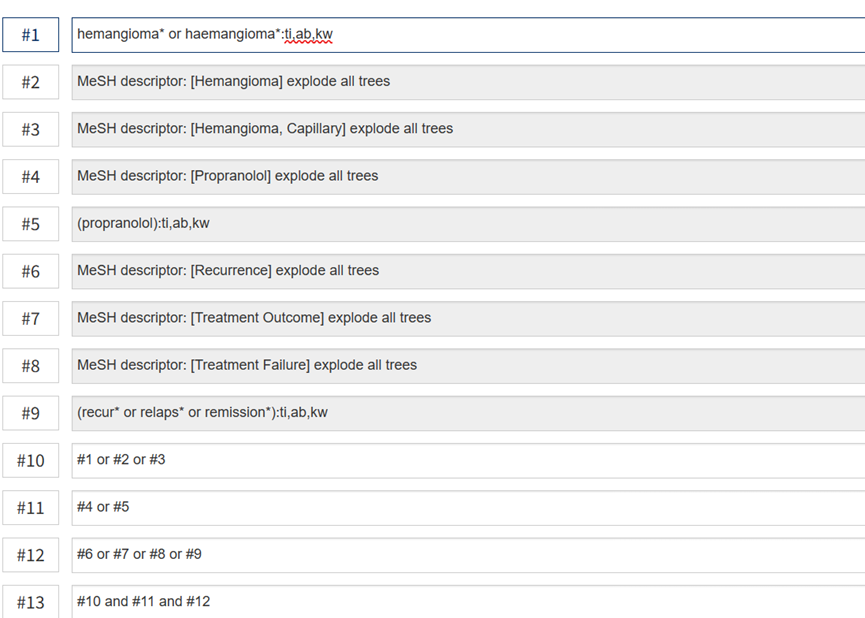


Pubmed :654（-2024.11.18）

(((hemangioma[MeSH Terms]) OR Hemangioma, Capillary[MeSH Terms] OR ((((hemangioma*[Title/Abstract]) OR hemangiomas[Title/Abstract]) OR haemangioma[Title/Abstract]) OR haemangiomas[Title/Abstract])))AND ((Recurrence[MeSH Terms]) OR (recur*[Title/Abstract] OR relaps*[Title/Abstract] OR remission*[Title/Abstract]) OR (treatment outcome OR treatment failure[MeSH Terms])) AND ((propranolol[MesH terms]) OR propranolol[Title/Abstract])

Web of science: 330（-2024.11.18）

1. **TI=hemangioma or TS=Hemangioma, Capillary or TI=haemangioma**
2. **TI=Recurrence OR TS=recur* OR TS=relaps* OR TS=remission* OR TS=treatment outcome OR TS=treatment failure**
3. **TI=propranolol**
4. **1 AND 2 AND 3**

Embase: 1005 （-2024.11.18）

| No. |  | Results |
| --- | --- | --- |
| #17 | #7 AND #13 AND #16 | 750 |
| #16 | #14 OR #15 | 102562 |
| #15 | propranolol ti,ab,kw | 0 |
| #14 | 'propranolol'/exp | 102562 |
| #13 | #8 OR #9 OR #10 OR #11 OR #12 | 3742842 |
| #12 | 'treatment outcome'/exp OR 'treatment failure'/exp | 2362606 |
| #11 | remission*:ti,ab,kw | 257635 |
| #10 | recur*:ti,ab,kw | 1099778 |
| #9 | relaps*:ti,ab,kw | 402404 |
| #8 | 'recurrence'/exp | 220021 |
| #7 | #1 OR #2 OR #3 OR #4 OR #5 OR #6 | 75905 |
| #6 | haemangiomas:ti,ab | 2110 |
| #5 | haemangioma:ti,ab | 3945 |
| #4 | hemangiomas:ti,ab | 11684 |
| #3 | hemangioma*:ti,ab,kw | 28994 |
| #2 | 'capillary hemangioma'/exp | 5236 |
| #1 | ('hemangioma'/exp OR hemangioma) AND 'hemangioma'/exp |  |

Clinical trial gov :17

Hemangioma, Capillary Infantile and propranolol
